# Low-cost inertial microfluidic device for microparticle separation: A laser-Ablated PMMA lab-on-a-chip approach without a cleanroom

**DOI:** 10.1016/j.ohx.2023.e00493

**Published:** 2023-11-11

**Authors:** Cristian F. Rodríguez, Paula Guzmán-Sastoque, Mónica Gantiva-Diaz, Saúl C. Gómez, Valentina Quezada, Carolina Muñoz-Camargo, Johann F. Osma, Luis H. Reyes, Juan C. Cruz

**Affiliations:** a Department of Biomedical Engineering, Universidad de los Andes, Cra. 1E No. 19a-40, Bogotá 111711, Colombia; b Department of Electrical and Electronic Engineering, Universidad de los Andes, Cra. 1E No. 19a-40, Bogotá 111711, Colombia; c Grupo de Diseño de Productos y Procesos (GDPP), Department of Chemical Engineering, Universidad de los Andes, Cra. 1E No. 19a-40, Bogotá 111711, Colombia

**Keywords:** Lab-on-a-chip, Microfluidic-device, Microparticle-separator, COMSOL, Low-cost

## Abstract

Although microparticles are frequently used in chemistry and biology, their effectiveness largely depends on the homogeneity of their particle size distribution. Microfluidic devices to separate and purify particles based on their size have been developed, but many require expensive cleanroom manufacturing processes. A cost-effective, passive microfluidic separator is presented, capable of efficiently sorting and purifying particles spanning the size range of 15 µm to 40 µm. Fabricated from Polymethyl Methacrylate (PMMA) substrates using laser ablation, this device circumvents the need for cleanroom facilities. Prior to fabrication, rigorous optimization of the device's design was carried out through computational simulations conducted in COMSOL Multiphysics. To gauge its performance, chitosan microparticles were employed as a test case. The results were notably promising, achieving a precision of 96.14 %. This quantitative metric underscores the device's precision and effectiveness in size-based particle separation.

This low-cost and accessible microfluidic separator offers a pragmatic solution for laboratories and researchers seeking precise control over particle sizes, without the constraints of expensive manufacturing environments. This innovation not only mitigates the limitations tied to traditional cleanroom-based fabrication but also widens the horizons for various applications within the realms of chemistry and biology.

## Graphical abstract

**Figure:**
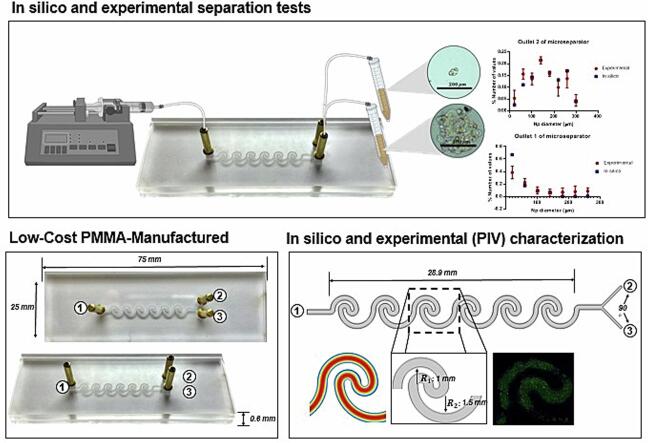

## Specifications table

Please replace the italicized instructions in the right column of the table with the relevant information about your hardware

**Table:** 

Hardware name	*Low-cost passive microfluidic separator*
Subject area	Engineering and materials science Chemistry and biochemistry Medical (e.g., pharmaceutical science)
Hardware type	*Other. Microparticle separator*
Closest commercial analog	*Microfluidic cell sorting system for example:* *CellRaft AIRE (Acoustic Inertial focusing for Rare Event)* (Bucher Biotec AG, Basel, Switzerland)
Open source license	*CC-BY 4.0*
Cost of hardware	*$0.661*
Source file repository	*https://doi.org/10.17632/yvspjzcn2h.1*

## Hardware in context

Micro and nanoparticles have gained significant recognition for their exceptional properties and numerous chemical and biological applications [1], [2], [3], [4], [5], [6]. Ensuring the homogeneity of these particles is of paramount importance to achieve optimal material performance. Addressing this necessity, microfluidic devices, also known as labs-on-a-chip (LOCs), have been developed to separate and purify particle suspensions [7], [8]. Based on the separation mechanism used, these LOCs can be classified as either active or passive separators [9], [10], [11]. Active LOCs utilize external forces, such as magnetic, ultrasonic, or electric fields [12], [13], [14], [15]. In contrast, passive LOCs rely solely on inter-particle forces, fluid properties, and channel geometry to separate particles [16]. Consequently, parameters such as fluid viscosity and inlet flow rate can have a significant impact on the accuracy of passive LOCs [17], [18].

Passive separators employ a variety of strategies, including inertial force, deterministic lateral displacement (DLD), microfiltration, and pinched flow fractionation (PFF) [19], [20], [21], [22], [23], [24]. Inertial forces capitalize on the varying inertial forces particles experience in a fluid flow with abrupt changes in direction [25], [26]. Larger, denser particles are pushed more forcefully toward the flow's deviated side, while smaller ones are displaced to a lesser degree [27], [28], [29]. This results in particle separation based on size or density difference, with the final deposition dependent on various factors, including the device wall, Reynolds number, particle deformability, size, density, and viscosity ratio [30], [31]. As per the findings by Dominic et al., there exists a clear correlation between particle size and particle separation efficiency. Notably, in the case of Ȧngström Sphere Soda-Lime Beads particles with a size of 120 µm managed to attain an almost flawless separation efficiency close to 100 % [32].

DLD, on the other hand, uses the motion of particles guided by their center of mass through laminar flow. This is accomplished by arranging a series of obstacles in the microchannel geometry, resulting in particle separation based on size [33], [34]. Larger particles encounter fewer interactions and progress along the channel, while smaller particles get displaced laterally due to interactions with the obstacles. In essence, DLD facilitates the sorting of particles by size within microfluidic systems, capitalizing on controlled obstacle-induced lateral motion [35], [36], [37]. Hyun et al., for instance, proposed a hybrid microfluidic platform that utilized a cascade deterministic lateral displacement matrix to separate MDA-MB-231 cells from blood samples, resulting in a recovery rate of nearly 93 % [38]. Microfiltration, which uses microporous membranes, is an additional technique employed in passive separators. This method separates particles by flow-through and typically consists of three components: an inlet weir, a membrane, and a final container. Dead or crossflow microfiltration is possible [39], [40]. For example, Fan et al. developed a polydimethylsiloxane (PDMS) membrane filter microdevice with a 90 % recovery rate for isolating circulating tumor cells from peripheral blood [41].

PFF separation relies on control of the flow rate ratio or asymmetry at the inlet of the flow outlet design. These devices typically exhibit T or Y junction shapes. In a PFF separator, two inlet streams are employed: one containing the particles to be separated and the other a buffer solution [42]. As these two streams converge at the microchannel's pinch point, a hydrodynamic force is generated, directing the particles toward a specific side of the channel [43], [44]. This process causes particles of different sizes to follow distinct flow trajectories [45], [46]. Chen et al. used this principle to develop a particle aggregation-based microseparator of polystyrene microspheres for DNA detection [45]. Berendsen et al. developed a microdevice to separate spermatozoa from an erythrocyte-filled biopsy sample collected from men with azoospermia, achieving a 95 % separation rate [47].

The domain of microfluidic particle separation has borne witness to the emergence of diverse and sophisticated techniques, each characterized by its distinct principles and advantages. Nevertheless, a shared limitation across these methodologies is their reliance on intricate manufacturing processes that are conventionally confined to controlled cleanroom settings. This prerequisite for meticulous fabrication often presents a hurdle for widespread adoption, thereby limiting accessibility for certain laboratories and researchers. In response to this challenge, we propose the development of an inertial microfluidic device employing laser ablation in Polymethyl Methacrylate (PMMA). This groundbreaking technique not only ensures rapid and cost-efficient production but also upholds exceptional separation efficiency.

## Hardware description

**Fig. 1:**
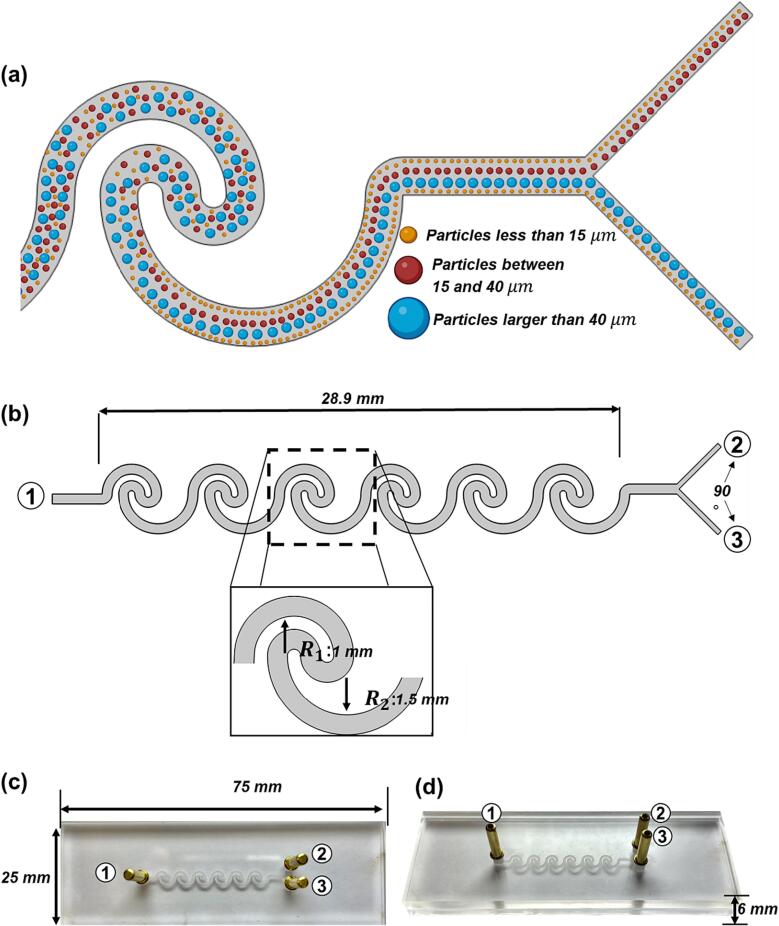
Microfluidic Device Geometry and Separation Mechanism (a) Microfluidic Device Separation Mechanism (Yellow: <15 μm, Red: 15–40 μm, Blue: <40 μm) (b) 2D sketch of the microchannel for the Multiphysics simulation of the microfluidic device. ①Solution inlet for purification ② Outlet for the smallest microparticles, ③ Outlet for the largest microparticles. (c-d) Actual picture of the low-cost microfluidic device manufactured using PMMA sheets and laser engraving. (For interpretation of the references to color in this figure legend, the reader is referred to the web version of this article.)

The microfluidic separator described here is a highly efficient device designed to separate particles between 15 and 40 µm in diameter. This design of the wave geometry has been rigorously validated in previous studies and has demonstrated its ability to effectively separate microparticles [48], [49], [50]. This partitioning relies on manipulating inertial forces within the complex channel geometry. Inertial forces utilize variations in fluid flow direction, compelling larger particles towards the deviated side while causing smaller ones to be displaced to a lesser extent. This inertial effect arises due to the interaction between fluid flow and particle motion in the wavy's intricate channel design. The unique pattern triggers distinct inertial forces acting on particles of different sizes, guiding their migration within the channels (Fig. 1a). The effect is enhanced through precise control of the volumetric flow rate, allowing customization of separation via inertial forces.

The device consists of a series of inverse corrugated channels that have an additive effect, enabling the separation of two subpopulations of particles, one ranging from 15 to 40 µm, while the other from 40 to 220 µm. The separator was fabricated using Polymethyl Methacrylate (PMMA) sheets of 75 mm by 25 mm, with a thickness of 6 mm, which is a low-cost and transparent material that allows visualizing easily the materials flowing inside the device Fig. 1b and c.

The configuration of the separator incorporates six wavys, each defined by specific dimensions, culminating in a total wavy distance of 28.9 mm Fig. 1d. The outlets of the wavys are strategically positioned with a 90-degree angular displacement, further enhancing the effectiveness of the separator Fig. 1c and d. With a precision of 96.14 % and a recall of 77.62 %, the elucidated separator provides a pragmatic and cost-effective solution for the challenge of particle separation within the specified size regime.

## Design files

### Design files summary

**Table:** 

Design file name	File type	Open-source license	Location of the file
*Blueprint*	CAD	CC-BY 4.0	*https://doi.org/10.17632/yvspjzcn2h.1*

## Bill of materials

### Bill of materials summary

**Table:** 

Designator	Component	Number	Cost per unit -USD	Total cost –USD	Source of materials	Material type
Microfluidic connectors	*Inlets and outlets*	3	$0.05	0.15	https://www.alibaba.com/product-detail/Custom-Deep-Drawn-Sheet-Metal-Stainless_1600530603447.html?spm = a2700.7735675.0.0.7c3dXhbvXhbvUu&s = p	Metal
Sheets of PMMA	Sheets of PMMA (7.5 cm x 2.5 cm)	2	$0.2555	$0.511	https://www.homedepot.com/b/Building-Materials-Glass-Plastic-Sheets-Plexiglass/N-5yc1vZc9x2	Polymer

## Build instructions

The microfluidic device for inertial passive particle separation was fabricated using a low-cost laser ablation technique that employs polymethyl methacrylate (PMMA) sheets as substrates, as described previously [51], [52]. The device's design was created in AutoCAD (AutoDesk Inc., Mill Valley, CA, USA) using parameters derived from the Multiphysics simulations conducted on COMSOL Multiphysics 6.1® software (COMSOL Inc, Stockholm, Sweden). Red and black were designated as the cutting and engraving lines, respectively.

**Fig. 2:**
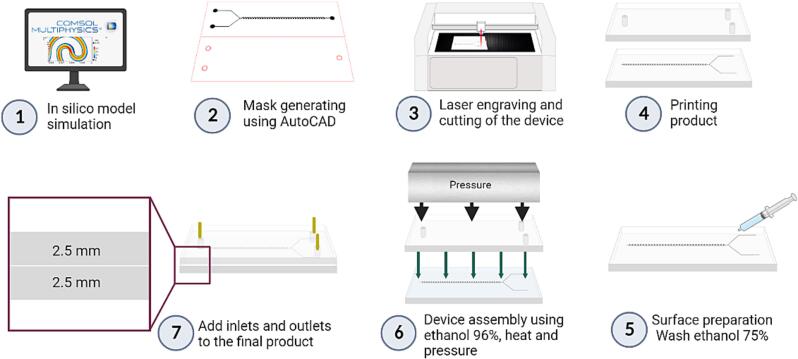
Manufacturing process for a microfluidic device based on PMMA sheets, a low-cost laser engraving technique and assisted by Multiphysics simulations. (1) Design optimization through Multiphysics simulations, (2) Engineering schematics of the cutting and engraving areas for subsequent laser cutting, (3) Laser engraving of the microfluidic device with appropriate speed and power for the desired channel depth, (4–6) Assembly of the bilayer PMMA device by gluing together sheets in the presence of 96 % (v/v) ethanol on a hot plate at 110 °C, while maintaining constant compression, (7) Attachment of the inlets and outlets aided by universal glue and curing at room temperature for 48 h.

The device was then fabricated utilizing a laser cutting system (TROTEC®, Marchtrenk, Austria), with a cutting speed of 0.5 and a power of 100 for the cutting process, and a speed of 10 and a power of 18 for the engraving one. After cutting and engraving, the PMMA was cleaned with 70 % (v/v) ethanolic solution. The PMMA sheets were then glued together using a thin layer of 96 % (v/v) ethanol and maintained under constant pressure at 110 °C for 10 min. The microfluidic device was completed by connecting its inlets and outlets. Fig. 2 illustrates the fabrication process.

## Operation instructions

**Fig. 3:**
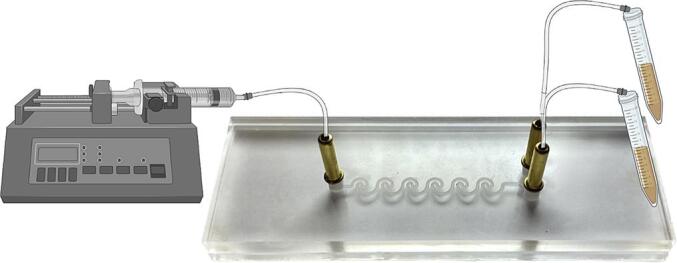
Operation of the microfluidic device using a syringe infusion pump. The first step involves connecting 15 cm tubes to the inlet and outlets of the device. The suspension to be purified was then pumped using a KDS-100 syringe infusion pump from W.P. Instruments (Holliston, MA, USA) at a flow rate of 23 mL/h. The purified suspensions were collected in two falcons located at the outputs of the device.

The operation of the microfluidic device for microparticle separation involves connecting 15 cm tubes to the device's inlet and outlets, followed by pumping the suspension to be purified into the device at a flow rate of 23 mL/h using a syringe infusion pump (KDS-100, W.P. Instruments, Holliston, MA, USA). The purified suspensions were then retrieved from two outputs on the device (Fig. 3).

## Validation and characterization

### Computational simulation

The software COMSOL Multiphysics 6.1® (COMSOL Inc, Stockholm, Sweden) was used to conduct an *in silico* evaluation using the particle tracing module. The fluid was considered under a laminar regime, governed by the Navier-Stokes equation for conservation of momentum (1) and the continuity equation for mass conservation (2).

(1) $$\rho \left(u\cdot \nabla \right)U=\nabla \cdot [-pI+\mu \left(\nabla U+{\left(\nabla U\right)}^{T}\right)]+F$$

(2) ρ∇·U=0

Where *u* is the fluid velocity, ρ is the fluid density, μ is the dynamic viscosity, *I* is the identity matrix, *p* is the fluid pressure, and *F* the external forces. The particle-tracing module was coupled to represent the microparticle movement along the channel, which is governed by the second law of Newton (3).

(3) $$F_{t}=\frac{d\left(m_{p}v\right)}{\mathrm{dt}}$$

Where $m_{p}$ is the mass of the particles, *v* is the velocity, and *F_t_* is the sum of all forces that affect the microparticles. The drag force (4) was also coupled to represent the parallel component of the force exerted by the fluid on the surface of the microparticles.

(4) $$F_{d}=\frac{1}{\tau_{p}}m_{p}\left(u-v\right)$$

Where *u* is the velocity field, $m_{p}$ is the particle mass, v is the particle velocity, and $\tau_{p}$ is the microparticle velocity response time, which is defined by equation (5).

(5) $$\tau_{p}=\frac{\rho_{p}d_{p}^{2}}{18\mu }$$

Where μ represents the fluid viscosity, $\rho_{p}$ the particle density, and $d_{p}$ the particle diameter. The lift force (6) was coupled to represent the perpendicular component of the force exerted by the fluid on the surface of the microparticles. This coupling holds particular significance within the context of microfluidic devices, where the lift force emerges as a decisive factor in dictating particle separation phenomena. [53], [54], [55]. It is noteworthy that this force distinctly exerts its greatest influence on particles that exhibit larger diameters and higher densities, shaping the intricate interplay of particle dynamics within such devices.

(6) $$F_{L}=\rho \frac{r_{\rho }^{4}}{D^{2}}\beta \left(\beta G_{1}\left(s\right)+\gamma G_{2}\left(s\right)\right)n$$

Where *ρ* is the particle density, $r_{\rho }$ is the particle radius, *D* represents the distance between the walls, *n* is the unit vector from the nearest point on the first parallel boundary, and *s* is the normalized distance to the first parallel boundary. $G_{1}$ and $G_{2}$ are coefficients in the function of wall distance given in [56], and β and γ are defined by equations (7), (8), respectively.

(7) $$\beta =\left|D\left(n\cdot \nabla \right)U_{p}\right|$$

(8) $$\gamma =\left|\frac{D^{2}}{2}{\left(n\cdot \nabla \right)}^{2}U_{p}\right|$$

Where *D* represents the distance between the channel walls, *n* is the wall-normal at the nearest point on the reference wall, and $U_{p}$ is defined by Eq. (9).

(9) $$U_{p}=(I-\left(n⊗n\right)u$$

Where *I* is the identity matrix, *n* is the wall-normal at the nearest point on the reference wall, and *u* is the fluid velocity.

**Fig. 4:**
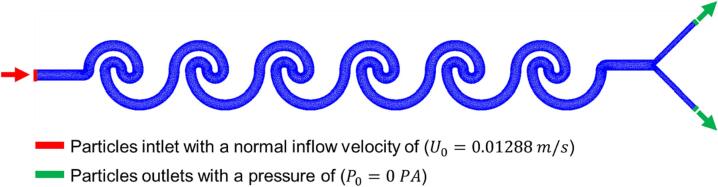
Computational domain and boundary conditions for the simulation. The mesh domain used in the simulation is shown along with the boundary conditions applied to the microfluidic device. The red color highlights the inlet, and the green color highlights the outlet of the microfluidic device. The imposed drag and lift forces act on the particles within the domain. (For interpretation of the references to color in this figure legend, the reader is referred to the web version of this article.)

A bidirectionally coupled particle tracing stud was employed with a MUMPS solver. As depicted in Fig. 4, the mesh configuration used in the simulations comprised 103,804 elements within the interior and 5,592 elements along its periphery, promoting model convergence, as evidenced in Fig. S1 of the supplementary materials. For the simulations, batches of 100 particles were systematically released into the computational domain in release ranges between 0 and 0.5 s, with intervals of 0.1 s.

**Fig. 5:**
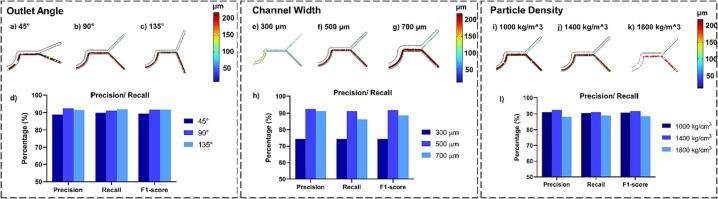
In-silico microfluidic performance analysis. (a-c) Particle Tracing Simulation Results for 45°, 90°, and 135° Outlet Angle. (d) Summary of Precision, Recall, and F1-Score for Outlet Angle Tests (45°, 90°, and 135°). (e-g) Particle Tracing Simulation Results for Channel Width test (300 µm, 500 µm, 700 µm). (d) Summary of Precision, Recall, and F1-Score for Channel Width test (300 µm, 500 µm, 700 µm). (i-k) Particle Tracing Simulation Results for particle density test (1000 kg/m^3^, 1400 kg/m^3^, 1800 kg/m^3^). (d) Summary of Precision, Recall, and F1-Score for particle density test (1000 kg/m^3^, 1400 kg/m^3^, 1800 kg/m^3^).

In silico simulations were employed to investigate the influence of varying outlet angles (45°, 90°, and 135°) on the performance of microdevices while keeping the parameters of particle density fixed at 1400 kg/m^3^ and the channel width at 500 µm. Fig. 5a illustrates the outcomes obtained with a 45° outlet angle, revealing a substantial adverse impact on separation precision. Specifically, Fig. 5d demonstrates a noticeable decline in precision, especially for particles falling within the 60 to 100 μm range, as Outlet 1 tends to select them.

Conversely, the utilization of a 135° outlet angle, as depicted in Fig. 5c, presents a different set of challenges. Simulations indicate an increased likelihood of particles becoming stuck in the outlet bifurcation of the device. Thus, it is evident from our simulations that the optimal outlet angle for these microdevices is 90°. Unlike the 45° angle, it does not compromise precision, and unlike the 135° angle, it avoids the risk of experimental tests leading to clogging.

Furthermore, we conducted *in silico* tests to evaluate the impact of channel width on the particle separation capacity of the microfluidic device while maintaining a fixed particle density of 1400 kg/m^3^ and an outlet angle of 90°. Three channel widths were explored: 300 µm, 500 µm, and 700 µm, illustrated in Fig. 5e – g, respectively. The investigation of the 300 µm channel width revealed a significant increase in the likelihood of particle entrapment within the device. Consequently, this channel width is unsuitable for separating particles with diameters exceeding 110 µm.

In contrast, the microfluidic device with a 700 µm wide channel exhibited a decrease in recall capacity, as evidenced in Fig. 5h, with numerous particles in the 60 to 150 µm diameter range erroneously classified through Output 1. The 500 µm channel width emerged as the optimal choice for the studied particle diameter range, delivering superior separation results. It reduced the occurrence of false negatives compared to the 700 µm width and mitigated the risk of obstructions observed with the 300 µm channel. This channel width outperformed others in maintaining precision and recall during particle separation.

Additionally, we explored the influence of particle density on the microfluidic device's separation performance through *in silico* tests using particles with densities of 1000 kg/m^3^, 1400 kg/m^3^, and 1800 kg/m^3^, as depicted in Fig. 5i – k, respectively. While keeping the outlet angle at 90° and the channel width at 500 µm constant. Low-density particles (1000 kg/m^3^) exhibited imprecise separation results, erroneously classifying several particles within the 60 to 120 µm diameter range as false negatives. This discrepancy was attributed to the insufficient inertia force generated by low-density particles, hindering their effective separation.

On the other hand, high-density particles (1800 kg/m3) also resulted in suboptimal precision and recall capacity, as observed in Fig. 5l. Several particles remained trapped within the device due to their increased density, leading to reduced performance. Our simulations conclusively demonstrated that the microfluidic device performed optimally with particles of intermediate densities, approximately 1400 kg/m3. This density range consistently delivered the most reliable and precise separation outcomes, effectively optimizing the device's performance.

In pursuit of microdevice optimization, the study focused on a configuration featuring a 90° outlet angle, a 500 µm channel width, and particles with a density of 1400 kg/m^3^. This customized microdevice underwent a thorough *in silico* analysis, resulting in the determination of critical parameters shedding light on its operational efficiency. The Mean Residence Time (MRT) was quantified at 4.431 s, representing the average duration particles spent within the device during separation. Moreover, the analysis revealed a Trajectory Length (TL) of 77.87 mm (mm), providing insight into the distance particles traveled while inside the device. Additionally, the Pressure Drop (P) was assessed at 55.04 Pa (Pa), offering valuable information about fluid pressure variations during particle separation within the device. These parameters, assessed under optimal conditions, collectively provide a comprehensive evaluation of the microdevice's performance, enhancing understanding of its capabilities and efficiency.

### Experimental tests

Three experimental tests were used to characterize and validate the microfluidic device: a Particle Image Velocimetry (PIV) system, a chitosan microparticle separation test, and an alginate microparticle separation test.

#### Particle Image Velocimetry (PIV)

Particle Image Velocimetry (PIV) was employed to elucidate the flow patterns within the device. To achieve this, fluorescent tracer particles containing Rhodamine B MV-F02 (Microvec, Piczów, Poland) were introduced into the device. Subsequently, the flow fields were visualized by capturing these microparticles with the aid of a PIV laser and isolating them from the surrounding elements using an induced fluorescence filter, MV-FILTER 550 (Microvec, Piczów, Poland).

**Fig. 6:**
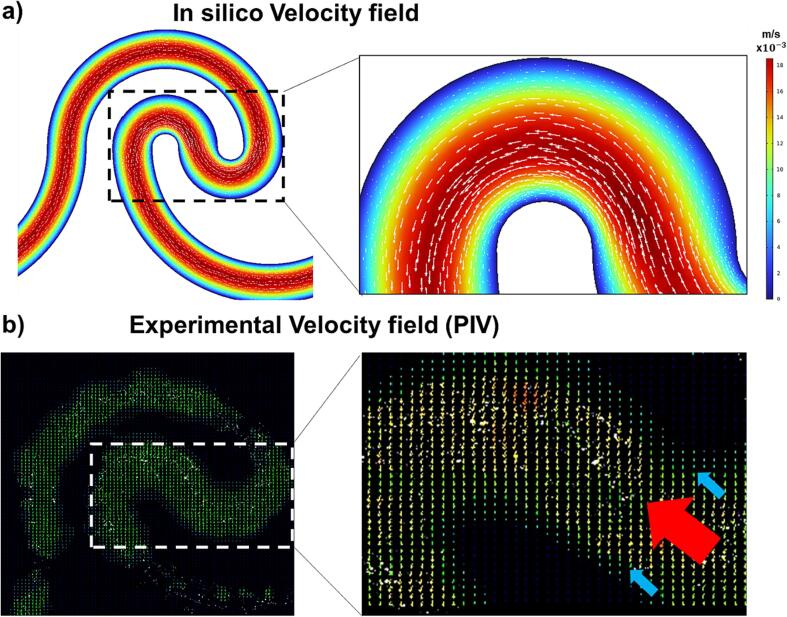
Microfluidic device characterization. (a) Computationally generated flow field using COMSOL Multiphysics 6.1® software (COMSOL Inc, Stockholm, Sweden). (b) Experimental validation of the flow field through Particle Image Velocimetry (PIV) measurements.

In Fig. 6, we present the characterization results obtained through PIV, which reveal a pronounced flow field effect within the device's curvatures. This phenomenon is attributed to the inertial forces generated by the geometry of the device. Both computational simulations (Fig. 6a) and experimental measurements (Fig. 6b) collectively underscore a consistent and distinct flow behavior within the microfluidic channel.

Specifically, our analysis demonstrates that the velocity distribution exhibits lower velocities in proximity to the channel walls and higher velocities in the central region of the channel. This discernible trend is visually represented by the blue arrows in the graphical depictions. This velocity distribution pattern implies the presence of a velocity gradient across the cross-section of the channel. Importantly, this variation in velocity directly influences the drag force experienced by the fluid within the microfluidic channel. The spatial changes in velocity are intrinsically linked to the distribution of drag forces along the length of the channel. The lower velocities near the walls contribute to diminished drag forces in those regions, while the elevated velocities in the central region correspondingly result in increased drag forces.

#### Chitosan microparticle separation

**Fig. 7:**
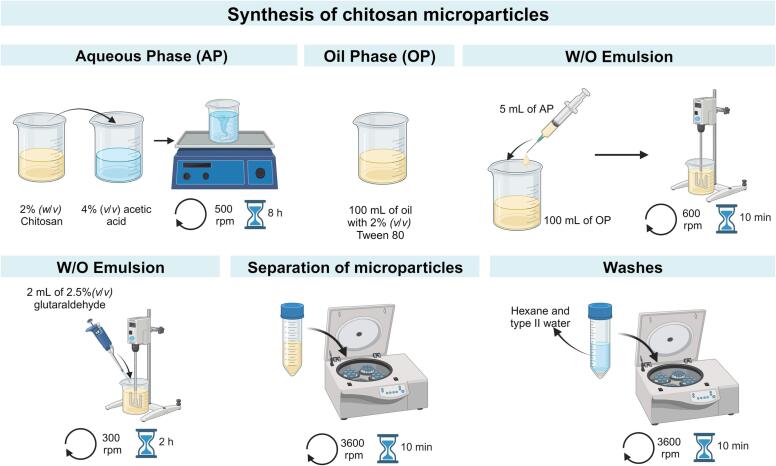
Protocol for the synthesis of Chitosan microparticles.

A chitosan microparticle separation test was conducted to validate the microfluidic device's efficiency further. A solution of chitosan microparticles was created by making variations to the protocol described by Mothilal et al. [57]. As shown in Fig. 7: Briefly, the oil phase was prepared with mineral oil and 2 % *(v/v)* Tween 80. The aqueous phase was prepared with a solution of 4 % *(v/v)* acetic acid and 2 % (w/v) chitosan, chitosan was dissolved in acetic acid under magnetic stirring at 500 rpm for 8 h. To make the W/O emulsion, 5 mL of the aqueous phase were added into 100 mL of the oil phase with a 22 G syringe. Then, the W/O emulsion was stirred for 10 min at 600 rpm through a mechanical stirrer Hei-TORQUE precision 200 (Heidolph, Schwabach, Germany). Next, 2 mL of 2.5 % *(v/v)* glutaraldehyde was added to the W/O emulsion and stirred at 300 rpm for 2 h. Finally, the microparticles were obtained by centrifugation at 3600 rpm for 10 min. Microparticles were washed with hexane and type I water (water with a resistivity > MΩ-cm and conductivity <1 µS/cm). The solution was then passed through the device’s inlet, and individual samples were collected at the device’s outlet. The particle size in each channel was analyzed using optical microscopy with the Zen 3.7® software (ZEISS, Jena, Germany).

**Fig. 8:**
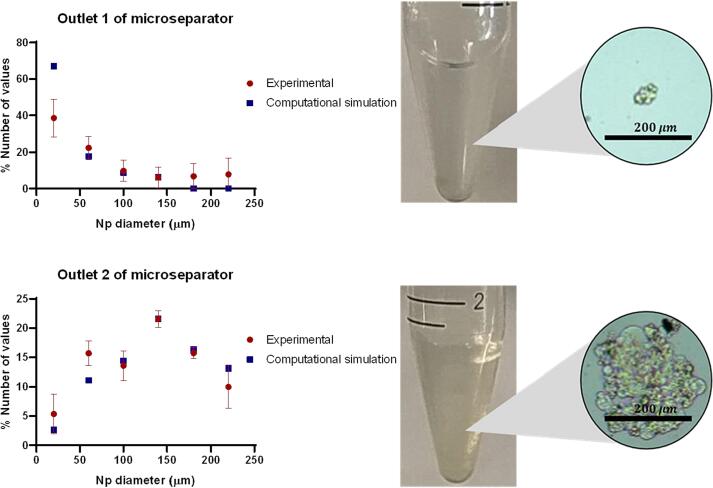
Separation efficiency of the microfluidic device. The *in silico* simulation and the experimental chitosan microparticle separation test conducted on the device are shown comparatively. The percentage of separated particles is shown on the Y-axis, while the particle size in micrometers is displayed on the X-axis.

The *in silico* and experimental separation results in Fig. 8 were assessed using a set of metrics aimed at appraising the performance of the microfluidic separator. These metrics, namely precision, recall, and the F1-score, collectively furnished insights into the device’s capability to accurately categorize particles based on their size attributes.

To facilitate the evaluation process, the following terminologies were employed:

True Positives (TP): This pertains to particles genuinely exceeding 40 µm in size that the separator adeptly identified and classified as such. In the context of the experiment, particles directed to outlet 2 aligned with this definition.

False Positives (FP): These denote particles possessing a size below 40 µm, erroneously labeled as surpassing the specified threshold by the separator. This category encompasses particles exiting through outlet 2, despite their dimensions falling short of the 40-µm criterion.

True Negatives (TN): Representing particles with dimensions below 40 µm, correctly identified as such by the separator. This class encapsulates particles channeled to outlet 1 that comply with the size requirement.

False Negatives (FN): Refers to particles exceeding 40 µm in size, yet inaccurately designated as being smaller than 40 µm by the separator. This category encompasses particles that ideally should have been directed to outlet 1 but were erroneously classified due to their size.

The ensuing metrics were computed as follows:

**Precision:** This parameter gauges the proportion of accurately identified particles (true positives) among the entire set of particles classified by the separator as exceeding 40 µm (true positives + false positives). It can be calculated as TP/(TP + FP). The computational outcome for this metric stood at **98.43 % *in silico*** (simulation-based analysis) and **96.14 % in experimental** evaluations.

**Recall** (Sensitivity or True Positive Rate): Recall evaluates the ratio of correctly recognized particles (true positives) against the aggregate of particles that genuinely possess dimensions exceeding 40 µm (true positives + false negatives). It can be expressed as TP/(TP + FN). The calculated values for this metric were **83.32 % *in silico*** and **77.62 % in experimental** investigations.

**F1-Score** (or the Harmonic Mean of Precision and Recall): The F1-score offers a unified metric that harmonizes both precision and recall considerations. Computed as 2 * (Precision * Recall)/(Precision + Recall), this metric resulted in values of **86.69 %** during ***in silico*** analyses and **79.70 % in experimental** scenarios.

#### Alginate capsule separation

**Fig. 9:**
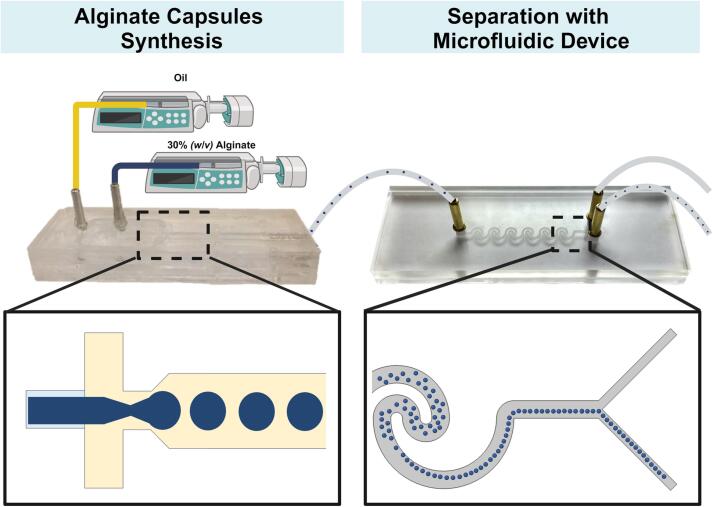
Alginate capsule separation test.

An alginate capsule separation test was conducted to validate the microfluidic device's efficiency further. Alginate capsules were synthesized with certain modifications to previously established protocols [58], [59]. To synthesize microparticles, we employed a microfluidic device, using a continuous phase of 30 % (w/v) sodium alginate and mineral oil as the dispersed phase. The flows were precisely controlled at 0.5 mL/h for the continuous phase and 300 mL/h for the dispersed phase using a syringe infusion pump (KDS-100, W.P. Instruments, Holliston, MA, USA). Following microparticle synthesis, the alginate droplets were introduced into the separation microfluidic device, as visually represented in Fig. 9. To prepare for the separation test, the coupled microfluidic devices underwent a stabilization process. For a detailed visual representation of this process, refer to Video S1 in the supplementary material. Following the completion of the stabilization process, the microfluidic device's inertia skillfully initiated the separation of droplets in outlet 2 with remarkable precision, as vividly demonstrated in Video S1 of the supplementary material. This validation test not only confirmed the device's proficiency in separating alginate capsules but also showcased its capacity to effectively separate various types of capsules. The separation was primarily guided by the size and density of the particles, underlining the versatility and adaptability of the microfluidic device for a wide range of applications. This revised section emphasizes the precision of the separation process and highlights the device's potential for handling different types of capsules, providing a more comprehensive perspective on its capabilities.

In conclusion, the developed microfluidic separator, fabricated from PMMA substrates using laser ablation and optimized through COMSOL Multiphysics simulations, demonstrated exceptional performance. Chitosan microparticles were successfully separated with a remarkable precision of 96.14 %, validating the device’s effectiveness.

While the current results are highly promising, future work aims to further enhance the device’s performance by improving the recall metric. This improvement will be pursued through modifications in the device’s geometry, particularly in the outlets, to ensure even more precise size-based particle separation.

This separator eliminates the barriers imposed by costly cleanroom facilities, making it an accessible and practical tool for laboratories and researchers seeking precise particle size control. Beyond its immediate applications, this technology opens doors to a myriad of possibilities in diverse fields, offering a versatile solution for size-based particle manipulation.

The research not only addresses the challenges associated with traditional fabrication methods but also contributes to the broader advancement of microfluidic technology. With its cost-effectiveness and robust performance, the presented microfluidic separator holds promise for accelerating research and applications in areas reliant on precise particle size control, including chemistry, biology, and beyond.

#### CRediT authorship contribution statement

**Cristian F. Rodríguez:** Conceptualization, Methodology, Software, Validation, Data curation, Writing – original draft, Visualization, Investigation, Writing – review & editing. **Paula Guzmán-Sastoque:** Conceptualization, Methodology, Software, Validation, Visualization, Investigation. **Mónica Gantiva-Diaz:** Conceptualization, Methodology, Software, Validation, Visualization, Investigation. **Saúl C. Gómez:** Conceptualization, Methodology, Software, Validation. **Valentina Quezada:** Conceptualization, Methodology, Software, Validation. **Carolina Muñoz-Camargo:** Supervision. **Johann F. Osma:** Conceptualization, Methodology, Software, Validation, Supervision. **Luis H. Reyes:** Supervision, Writing – review & editing. **Juan C. Cruz:** Conceptualization, Methodology, Software, Validation, Supervision, Writing – review & editing.

## Declaration of Competing Interest

The authors declare that they have no known competing financial interests or personal relationships that could have appeared to influence the work reported in this paper.
